# Implementation of soluble CD38 in blood crossmatching for patients on anti‐CD38 therapy: A single‐center experience

**DOI:** 10.1111/trf.70389

**Published:** 2026-09-11

**Authors:** Ayda Javanbakht, David Hanna, Grishma Amatya Jaspau, Ravi Sarode

**Affiliations:** ^1^ Department of Pathology University of Texas Southwestern Dallas Texas USA; ^2^ Transfusion Services University of Texas Southwestern Dallas Texas USA; ^3^ Department of Internal Medicine University of Texas Southwestern Dallas Texas USA

**Keywords:** anti‐CD38, crossmatch, soluble CD38

## Abstract

**Background:**

Human monoclonal (MoAb) anti‐CD38 antibodies, such as daratumumab, are used to treat diseases such as multiple myeloma. Because red blood cells (RBCs) express CD38, these MoAbs interfere with antibody screening and crossmatching in blood banks. The dithiothreitol (DTT) treatment of reagent cells to destroy CD38, combined with phenotyping/genotyping, has been used to address this interference. Due to anti‐CD38 activity in the patient's plasma, RBC units will be crossmatch incompatible and labeled crossmatch‐incompatible, necessitating communication with the ordering team regarding the safety of blood transfusion. The recombinant soluble CD38 (sCD38) antigen neutralizes the MoAb in a patient's plasma and can be used to perform crossmatches. Here, we describe our experience with sCD38 in RBC crossmatching.

**Study Design and Method:**

We began using sCD38 (Grifols) for RBC crossmatching in January 2026 (using 6 μL of sCD38 per crossmatch) following completion of a validation study. Because of the known interference of sCD38 with the Duffy blood group system (by denaturation), its use was limited to RBC crossmatching. We use the DTT method for antibody screening tests.

**Results:**

A total of 128 RBC crossmatches were performed for 49 patients on daratumumab. The sCD38 neutralized daratumumab in all crossmatching samples. Thus, all units were labeled crossmatch‐compatible, eliminating the need for provider communication. At the last follow‐up, with type and screen for these patients, no new alloantibody was detected.

**Conclusion:**

Soluble CD38 neutralized the anti‐CD38 MoAb, enabling crossmatch‐compatible RBC units to be issued and obviating the need to notify the provider of apparent incompatibility.

**Abbreviations**
DTT: dithiothreitol
EMR: electronic medical record
MM: multiple myeloma
MoAb: monoclonal antibody
RBC: red blood cell
sCD38: soluble CD38

## 1 INTRODUCTION

The CD38 antigen is a transmembrane glycoprotein expressed on various cell types, including red blood cells (RBCs), plasma cells, smooth muscle cells, and malignant cells such as plasma cells in multiple myeloma (MM). It plays a role in immune cell activation, adhesion, calcium signaling regulation, and energy metabolism.^1^

Therapeutic monoclonal antibodies (MoAbs), such as anti‐CD38, target CD38 on the surface of immune and myeloma cells. Two US Food and Drug Administration‐approved anti‐CD38 monoclonals, daratumumab and isatuximab, are used to treat patients with relapsed, refractory, or newly diagnosed MM.^2^ Daratumumab is the first‐line therapy in newly diagnosed MM patients. Other anti‐CD38 medications include MOR202 and TAK‐079, which are mostly used in Europe and Japan.^3^ The use of anti‐CD38 MoAb therapies has expanded significantly. Numerous studies are evaluating their effects in other conditions, including cold agglutinin disease, warm autoimmune hemolytic anemia, and rheumatoid arthritis.^4^, ^5^, ^6^

Anti‐CD38 MoAbs interfere with blood bank tests, including antibody screening (indirect antiglobulin), antibody identification, and crossmatching, because CD38 expression on RBCs causes panagglutination. This interference could mask underlying alloantibodies and increase the time and effort required to provide RBC units for transfusion.^7^ These MoAbs do not interfere with the ABO blood typing.

Several approaches have been implemented to mitigate this interference by the anti‐CD38 in the blood bank testing. First, patients' RBCs are either phenotyped (before starting the therapy) or genotyped. Second, the use of dithiothreitol (DTT) to destroy the CD38 antigen expressed on screening RBCs by disrupting the disulfide bonds.^8^ However, DTT treatment also destroys Kell antigens; therefore, Kell‐negative RBCs are provided to patients if they are Kell negative or the Kell type is unknown.^9^ Because the patient's initial antibody screen is generally positive, the blood bank laboratory information system requires a physical crossmatching by indirect antiglobulin test of the unit before dispensing. Since these MoAbs interact with donor RBCs, the crossmatches are incompatible and labeled as crossmatch incompatible. This requires timely communication and explanation with providers and nurses, as well as documentation in the electronic medical record (EMR). These steps require significant time and effort (average 45 min), especially at night and on weekends, which can cause confusion among cross‐covering physicians and nursing staff on the floor.

Recently, a recombinant soluble CD38 (sCD38) antigen reagent that neutralizes anti‐CD38 MoAb in plasma has been introduced by Medion Grifols Diagnostics (Düdingen, Switzerland). According to the manufacturer, antibodies against Duffy antigens may react weaker or negatively after sCD38 treatment (the exact mechanism of Duffy antigen loss is still unknown); therefore, it may not be suitable for a patient's antibody screening test unless the patient's Duffy phenotype or genotype is known and Duffy‐matched or Duffy‐negative RBCs are provided.^10^

In this brief report, we describe our strategy for managing patients receiving these MoAbs and our experience with sCD38 in RBC crossmatching.

## 2 MATERIALS AND METHODS

Before the use of sCD38, an RBC genotype was required before patients were started on anti‐CD38 MoAb (e.g., daratumumab). Because the DTT method is used for antibody screening—and DTT interferes with the Kell antigen—we provided Kell phenotype/genotype–matched RBC. Due to interference from anti‐CD38 MoAb, crossmatches were incompatible, and a pathologist was notified to contact the ordering provider and document it in the patient's EMR.

Validation of the new reagent for RBC crossmatching: Our sCD38 method involves pipetting 6 μL of sCD38 reagent (Grifols) into a 12 × 75 mm glass tube, adding 25 μL of the patient's plasma, and incubating at 37°C for 15 min. Then 50 μL of 0.8% donor red cells are pipetted into the anti‐IgG microtube of a gel card, followed by 25 μL of the sCD38‐treated plasma. The gel card is then incubated at 37°C for 15 min, centrifuged in the DG Spin for 9 min, and read.

We included 40 specimens containing anti‐CD38 (daratumumab) and performed manual crossmatches. These specimens were spiked with monoclonal phenotyping reagent alloantibodies, including anti‐^E^, anti‐^C^, anti‐^c^, anti‐^e^, anti‐^K^, anti‐^Jk^ ^a^, anti‐^Fy^ ^a^, anti‐^Fy^ ^b^, anti‐S, and anti‐N. Our acceptable criteria were that anti‐CD38 reactivity should be completely neutralized in all specimens treated with sCD38 and expected clinically significant alloantibodies should still be present in all specimens treated with sCD38, except for the Duffy group. Since we perform antibody screening using the DTT method, any Duffy group alloantibody should be detected, and antigen‐negative blood would be provided. During validation, all crossmatches were compatible; underlying alloantibodies (anti‐C, anti‐c, anti‐E, anti‐K, anti‐Jk^a^, anti‐S, and anti‐N) were unaffected (as expected, Fy^a^ and Fy^b^ antibodies were not detected), and validation was deemed successful.

We implemented the use of sCD38 for cross‐matching donor units in January 2026 in patients on anti‐CD38 therapy (daratumumab). Because of the known interference of sCD38 with the Duffy blood group, we continue to use the DTT method for antibody screening. If the patient makes anti‐K, this will appear as a crossmatch incompatible RBC unit with sCD38 treatment. We will then use sCD38‐treated plasma to identify anti‐K and provide compatible RBC units.

The institutional review board approved the retrospective analysis of patient data.

## 3 RESULTS

Forty‐nine patients (59.1% male) with an average age of 67 years (29–88 years) on anti‐CD38 MoAb who underwent 128 crossmatches using sCD38 between January and June 2026 were included in this study. The DTT method was used for the antibody screen test. One patient had a prior anti‐E antibody, and the rest had negative antibody screen results. All of them had red cell genotyping performed.

After implementing sCD38 treatment, all anti‐CD38 MoAb were completely neutralized in the patients' plasmas. The average timing of last dosage of daratumumab to cross match was 21 days (2–100 days). All RBC units were cross‐matched, compatible, and labeled as such, eliminating the need to contact floor providers and saving time for both pathologists and the clinical team (Table 1). Also, we did not provide K‐negative units. None of these patients have developed any new alloantibodies so far, as of the last type and screen (2–4 weeks) from the last transfusion.

**TABLE 1:** Comparison of blood crossmatching with and without sCD38.

	No sCD38 X‐match	sCD38 X‐match
DTT treatment	Yes	Yes
Reagent cost per one unit	$0	$13.78
Kell—matching (cost per one unit)	Yes‐ $54 (mandatory)	No‐ $0
Crossmatch labeling	Incompatible	Compatible
RBC phenotype/genotype cost	$350	0
Average blood bank technician and pathologists time needed for effective communication/documentation	45 min	0 min

Abbreviations: DTT, dithiothretol; RBC, red blood cell; sCD38, soluble CD38; X‐match, crossmatch.

## 4 DISCUSSION

In our validation study, followed by real‐life patients on anti‐CD38 MoAb therapy, we showed that 6 μL sCD38 completely neutralized daratumumab, allowing us to perform crossmatches without interference, including one patient with pre‐existing anti‐E. This process eliminated the need to provide K‐negative units, label donor units as incompatible, and involve pathologists. Thus, it improved the workflow in the transfusion service and provider satisfaction.

In a multicenter study in Europe, they used sCD38 for antibody screening of 273 patient samples. They evaluated the amount of sCD38 required to neutralize CD38 MoAb and found that 6 μL was sufficient to achieve complete inhibition in 98.5% of specimens (269 of 273). In their study, sCD38 failed to detect anti‐Fy^a^ and Fy^b^ alloantibodies in 10 different samples. Because of their concern for Duffy alloantibodies, they provided Duffy genotype‐matched RBC to their patients.^10^ They focused on the use of sCD38 for antibody screening tests and crossmatching.^10^, ^11^

Given the challenges of supplying Duffy phenotype‐matched RBC units and the associated costs, we opted to continue performing antibody screening using the DTT method. To the best of our knowledge, we are the first center to combine the DTT method for antibody screening followed by the sCD38 neutralization method for crossmatching to identify compatible RBC units in patients receiving daratumumab. This approach avoids the need to provide K‐negative RBC units and to label RBC units as incompatible. Thus, it also eliminates unnecessary phone calls and EMR documentation explaining the safety of units that would have been labeled incompatible.

The strength of the study is that we tested 128 specimens with sCD38 MoAb and consistently identified cross‐matched compatible RBC units. We also eliminated the need to genotype or phenotype patients eligible for daratumumab therapy. A limitation of this study is that it is a single‐center experience involving 51 patients and assessed sCD38 only in patients treated with daratumumab. We did not have any patients on other anti‐CD38 therapies, such as isatuximab.

In conclusion, sCD38 can completely neutralize MoAb anti‐CD38 in plasma from patients on daratumumab and facilitate RBC crossmatching. This approach provides compatible RBC units and avoids unnecessary steps related to spuriously incompatible units.

## CONFLICT OF INTEREST STATEMENT

The authors have disclosed no conflicts of interest.

## Data Availability

The data that support the findings of this study are available on request from the corresponding author. The data are not publicly available due to privacy or ethical restrictions.
